# When Air Leaks Meet Innovation: Obturator-Assisted Non-invasive Ventilation in a Preterm Infant With a Unilateral Cleft Lip and Palate

**DOI:** 10.7759/cureus.113110

**Published:** 2026-07-21

**Authors:** Matthew Michienzi, Abigale Cox, Patricia M Walworth, Katrina Savioli

**Affiliations:** 1 Pediatrics and Neonatology, Brooke Army Medical Center, San Antonio, USA; 2 Maxillofacial Prosthodontics, Brooke Army Medical Center, San Antonio, USA

**Keywords:** difficult airway management, extubation failure, maxillofacial prosthesis, nasoalveolar molding (nam), neonatal airway management, neonatal intensive care unit (nicu), non-invasive positive pressure ventilation, palatal obturator, premature infants, unilateral cleft lip and palate

## Abstract

Preterm infants often have respiratory distress requiring non-invasive respiratory support. Those born with a cleft lip and palate pose significant challenges in maintaining positive pressure due to anatomic differences that can cause substantial air leak when using typical mask/cannula interfaces. This leads to difficulties with ventilation and oxygenation for these infants whose lung disease would otherwise not necessitate invasive ventilation.

The purpose of this case report is to provide a multidisciplinary approach to a preterm infant with a unilateral cleft lip and palate for innovative solutions in respiratory support, allowing the best possible patient outcomes by minimizing the days requiring endotracheal intubation.

A 28+1-week male infant with a birth weight of 1540 g and prenatally diagnosed left-sided cleft lip and palate was born prematurely for maternal reasons. There were difficulties supporting the infant via non-invasive ventilatory methods, and multiple intubation attempts were required due to underlying anatomic airway anomalies. Upon literature review and discussion with the maxillofacial prosthetics team at our institution, a custom oral obturator was developed to provide a pseudo-palate for this infant. This allowed for decreased air leak and facilitated successful extubation to non-invasive positive pressure ventilation (NIPPV) with a RAM cannula interface (Neotech Products LLC, Valencia, CA, USA). Three evolving versions of the obturator were developed, with the final version that provided the best seal, reduced dislodgement rates, decreased oral ulcerations, and overall improved infant comfort.

Use of a custom oral obturator in preterm infants to facilitate extubation to NIPPV is feasible early in the clinical course. A multidisciplinary care team is required that includes maxillofacial prosthetics or a similar specialty that can mold this type of device. This can provide a framework for future design and use of an oral obturator to facilitate earlier extubation and minimize long-term complications associated with prolonged intubation in the neonatal period.

## Introduction

Infants born prematurely may require varying levels of respiratory management depending on the degree of lung development, with a direct relationship between lung maturity and gestational age. More commonly, the underlying pathology is respiratory distress syndrome, with many neonatal intensive care units (NICUs) commonly maintaining positive end-expiratory pressure through 32-34 weeks postmenstrual age [1]. In an infant who is born before 32 weeks' gestational age, the need for positive pressure to support their lung development may be further complicated when there are anatomic variants such as a cleft lip and palate.

Depending on the severity of the defect, there are challenges in utilizing typical non-invasive respiratory modalities (i.e., bubble continuous positive airway pressure (bCPAP) with mask or nasal prong interfaces) to support this patient population. Pesun et al. developed a custom oral obturator fixated to a nasal continuous positive airway pressure (CPAP) device to minimize air leak through the defect and ultimately facilitate extubation to a non-invasive modality at 34 weeks of gestation [2].

This case highlights the successful use of a subspecialist not frequently utilized in the NICU to create a custom dental obturator with a proper seal at one week of life for a 28+1-week infant born with a significant left-sided cleft lip and palate. This allowed for extubation much sooner than described by Pesun et al. to avoid the bronchopulmonary dysplasia diagnosis and complications of prolonged intubations [2-5]. 

## Case presentation

A 1540-g male infant was born at 28+1 weeks’ gestation via cesarean section to a 23-year-old gravida 2, para 1 (G2P1) mother with chronic hypertension and superimposed preeclampsia. Prenatal ultrasound identified a cleft lip and palate, and antenatal betamethasone was administered prior to delivery. Delivery was uncomplicated with delayed cord clamping and Apgar scores of 8 and 8 at one and five minutes, respectively.

Due to prematurity, the infant was placed on bCPAP shortly after birth but later required surfactant and intubation for worsening oxygenation. Airway management proved difficult, requiring six attempts across multiple providers before successful intubation by pediatric otolaryngology using video laryngoscopy. Given concern for airway edema after repeated attempts, the infant remained intubated on low ventilator settings. An extubation attempt at 48 hours of life to non-invasive positive pressure ventilation (NIPPV) via nasal mask resulted in respiratory acidosis and inability to maintain adequate inspiratory pressures, necessitating reintubation. One week later, accidental self-extubation occurred. Due to the poor seal related to the cleft defect, the infant was unable to sustain ventilation with NIPPV despite trials with RAM cannula (Neotech Products LLC, Valencia, CA, USA) and modified CPAP mask interfaces. This resulted in repeat intubation.

Maxillofacial prosthetics was subsequently consulted, and a custom oral obturator was recommended to function as a pseudo-palate to reduce air leak through the cleft defect and allow effective non-invasive respiratory support. The infant was transitioned to NIPPV with the obturator at 29+4 weeks corrected gestation and 1462 g (stable and anticipated weight loss over the first week of life for a premature infant), earlier and at a lower weight than in previously reported cases, in an effort to minimize complications of prolonged invasive ventilation.

Initial obturator designs were complicated by frequent dislodgement and oral ulceration related to palate fit and external metal support wires, occasionally resulting in bradycardia and desaturation events that resolved with repositioning and temporary mask CPAP. Modifications to the device improved fixation but did not fully resolve pressure-related ulcers. A second, lower-profile obturator, inspired by nasoalveolar molding (NAM) plates, made of softer material, eliminated dislodgement and allowed prior oral ulcers to heal while improving patient comfort. Following successful support with this device, the infant was weaned from respiratory support by 34 weeks’ corrected gestation.

Materials and methods

The maxillofacial prosthetics team made several iterations of a custom oral obturator. Initially, an intraoral impression of the maxillary arch was made using Aquasil Ultra+ Smart Wetting Fast Set Putty (Dentsply Sirona, Charlotte, NC, USA). Prior to impressing, a test was performed to determine the optimal mixing time of the material to ensure an adequate impression with the shortest intraoral setting time due to the inability to provide airflow to the patient during the procedure. Mixing for 2:15 provided a malleable material that set in 30 seconds, which was deemed acceptable by the provider team. The putty was mixed and attached to the handle of an infant impression tray, as the tray itself was too large to insert into the patient’s mouth. The impression was made and poured in type V dental stone (Die-Keen® Green, Modern Materials; Kulzer, Inc., South Bend, IN, USA) to produce the master cast.

**Figure 1:**
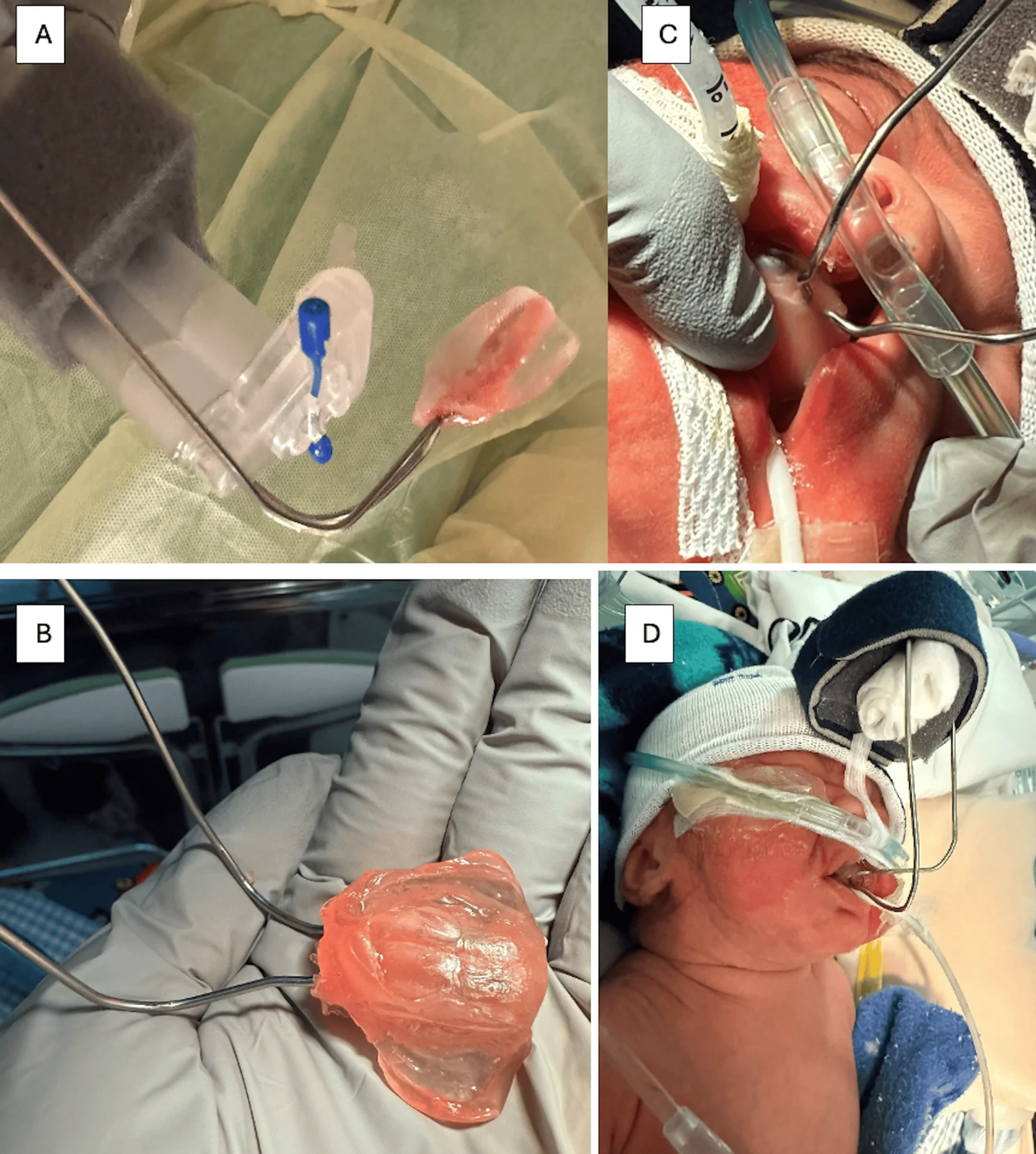
A-B) Version one of an oral obturator device attached to a nasal bubble continuous positive airway pressure device. C) The device was fixated in the patient while intubated, and the RAM cannula device (Neotech Products LLC, Valencia, CA, USA) was in place. D) The device was fixated with the RAM cannula in place after extubation. Note: The parents of the patient consented to have the patient's images used in an open-access publication. A written and signed consent statement was provided to the journal.

An acrylic obturator base was fabricated from clear ProBase® Cold Cure acrylic (Ivoclar Vivadent AG, Schaan, Liechtenstein) and was cured in a pressure pot. A channel was added to the cameo surface in the mid-palate of the base to allow attachment of a wire to aid in obturator retention. A 0.051-inch gauge stainless steel wire (Great Lakes Dental Technologies, Tonawanda, NY, USA) was pre-bent to fit around the FlexiTrunk™ nasal CPAP interface (Flexicare Medical Ltd., Mountain Ash, Wales, UK). The wire was designed to sit between the tubes of this interface, allowing ease of removal for obturator adjustments and replacement of mask components as needed (Figures 1a, 1b).

With the patient intubated and ventilated, the obturator base was inserted in the mouth, and a passive seat without areas of blanching was observed through the prosthesis. The base was relined with Shoreliner® Tough Silicone Denture Liner material (Tokuyama Dental Corporation, Tokyo, Japan) to improve the closure of the defect, especially around the cleft lip and anterior palate. The initial prosthesis was then assembled in the patient’s mouth to obtain the proper angulation to obturate the defect and have maximum retention from the wire. The mask was placed on the patient’s nose with the wire positioned between the tubes and attached to the obturator using Stellar DC Acrylic Pickup Resin (Taub Products and Dental, Inc., Melville, NY, USA). The excess wire was trimmed outside the mouth, and the prosthesis was polished. The prosthesis was then inserted into the patient’s mouth. The prosthesis was able to be fully seated in the cleft and provided a good seal; however, placing the wire attachment around the tubing caused dislodgement of the obturator, which would lead to air leakage when fully assembled. The wire loop was cut and bent to better provide simultaneous retention and a seal and was secured to the FlexiTrunk™ nasal CPAP interface (Figure 1c). The patient’s lip was then taped with a steri-strip to provide a bridge over the external cleft, the RAM cannula was placed and secured, and the patient was extubated to NIPPV.

Over the next 24 hours, the obturator was dislodging with head movement, leading to multiple desaturation episodes. Additionally, several pressure ulcers formed on the patient’s ridge and in the anterior cleft palate. The intaglio of the obturator was adjusted in pressure areas using a straight handpiece and acrylic dental burs. Another prosthesis was fabricated in the same fashion with the wires bent at a different angle to allow more passive attachment to the FlexiTrunk™ nasal CPAP interface (Figure 1d). The prosthesis was relined with Shoreliner Tough Silicone Denture Liner, and the intraoral seal was confirmed when the device was fully assembled. The prosthesis appeared to have better retention; however, the seal was again disrupted with any movement of the patient’s head, leading to an increased number of desaturations.

**Figure 2:**
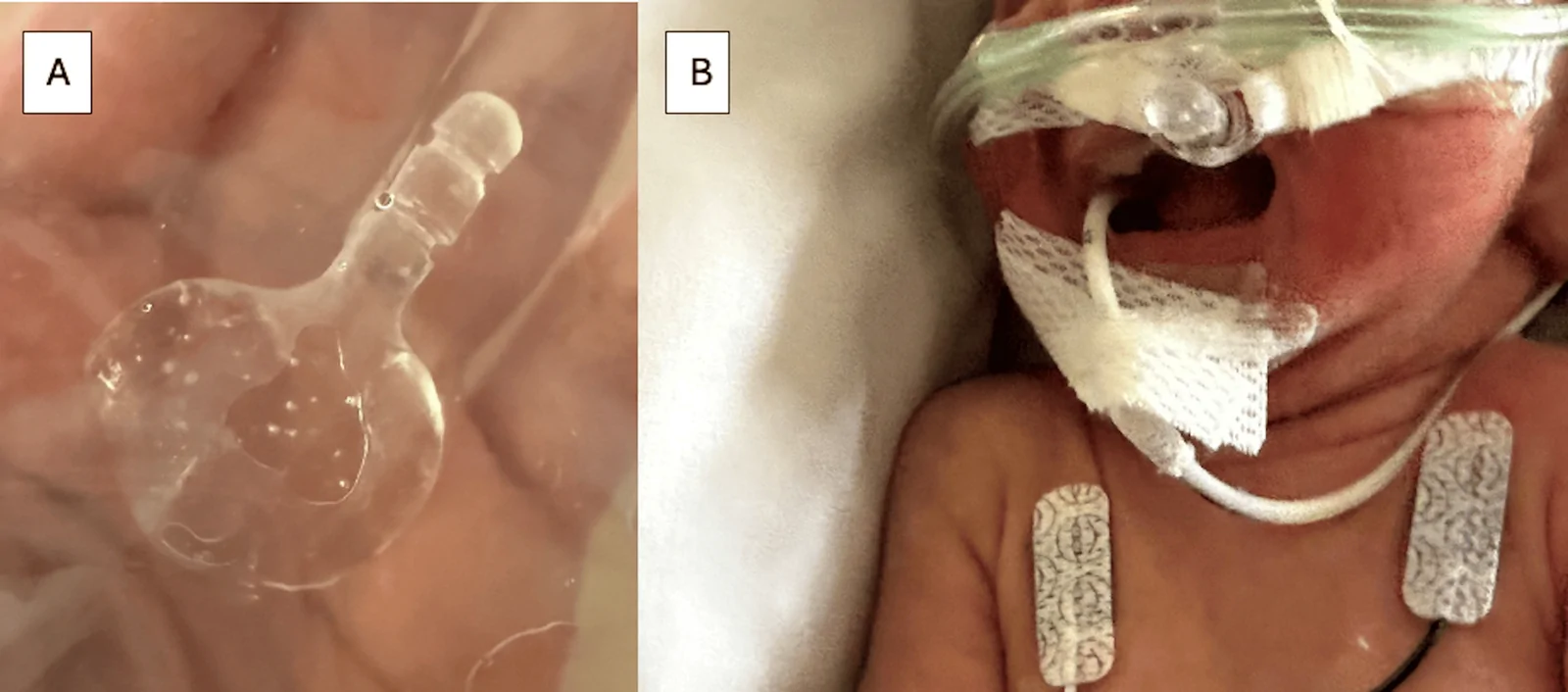
A.) Version two of the obturator device. B.) Version two of the oral obturator device with the accompanying nasal RAM cannula (Neotech Products LLC, Valencia, CA, USA).

A second version of the oral obturator was designed to eliminate the wire and head strap assembly. The design was similar to a NAM device utilized in cleft palate infants to approximate cleft segments prior to surgical correction [6]. The stone model was modified to allow the addition of a button in the anterior of the obturator base in the area of the cleft lip. The button was poured in clear ProBase® Cold Cure acrylic in conjunction with base fabrication, and the prosthesis was cured in a pressure pot. Two grooves were added circumferentially around the button to facilitate prosthesis retention (Figure 2a).

The obturator base was inserted into the patient’s mouth, and even tissue pressure was observed through the prosthesis. The base was relined with Coe-Soft® Resilient Denture Liner (GC America Inc., Alsip, IL, USA) to provide increased intraoral retention and a seal in the cleft lip. Steri-Strips™ were then secured around orthodontic elastic bands (3M™ Unitek™ Elastics, ¼-inch Medium, 4 oz; 3M Oral Care, Monrovia, CA, USA) and cross-taped to ensure that the bands would not dislodge. Base tape was applied to the patient’s cheek. The elastic bands were placed into the groove on the button, and a light stretch was applied prior to securing the steri-strip to the base tape bilaterally. Equal tension on the right and left bands was ensured to retain the prosthesis but not force movement of the cleft segments. Adequate seal of the defect was observed and reflected in oxygen saturation levels, and little to no movement of the obturator was detected (Figure 2b).

Follow-up was performed at 24 hours, 72 hours, and five days post initiation of the second version of the oral obturator to ensure an adequate seal and no pressure ulcers. Two ulcerations developed roughly at 72 hours. The intaglio of the prosthesis was adjusted, and the ulcers resolved within three days. The patient was examined daily to check for ulcers and weekly for the need to reline the prosthesis due to growth.

## Discussion

This case demonstrates the successful use of a custom oral obturator to facilitate non-invasive respiratory support in a very preterm infant with cleft lip and palate who otherwise required prolonged invasive ventilation due to an inability to maintain an adequate seal with conventional non-invasive interfaces [7]. By functioning as a pseudo-palate and reducing air leak through the cleft defect, the obturator enabled effective NIPPV and allowed earlier transition away from endotracheal intubation. This approach is clinically important because prolonged intubation in premature infants is associated with complications, including subglottic stenosis, ventilator-associated pneumonia, airway trauma, and oral aversion [3]. Additionally, reducing prolonged intubation may support earlier progression toward oral feeding, which is often delayed in premature infants and can be further complicated by cleft lip and palate [8-9]. Our case highlights how collaboration with maxillofacial prosthetics to create a customized obturator may provide a practical strategy to overcome limitations of conventional non-invasive ventilation in this high-risk population.

**Figure 3:**
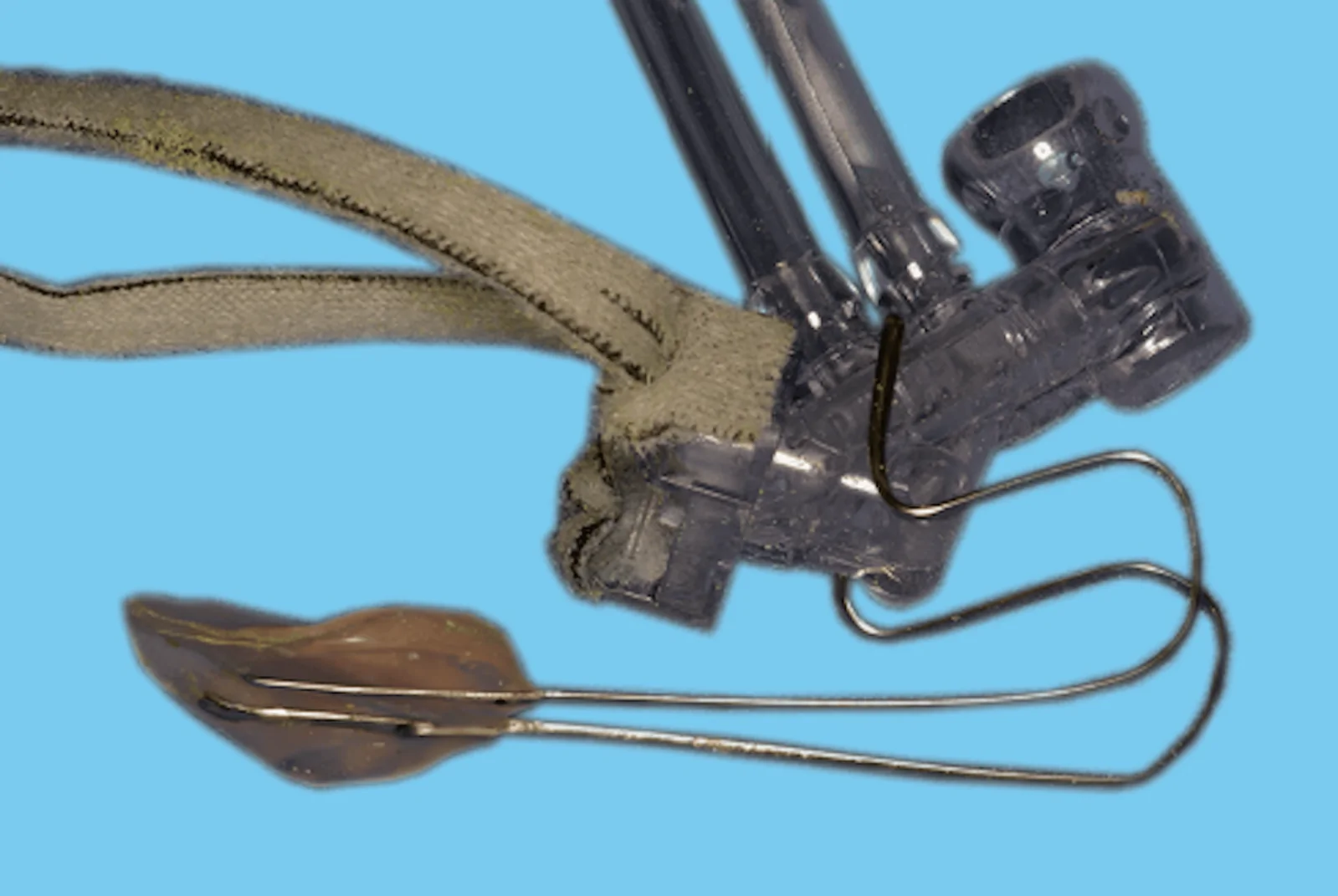
Image of the oral obturator attached to the nasal continuous positive airway pressure device. Reproduced from The Journal of Prosthetic Dentistry, Vol. 113, Pesun IJ, Minski J, Narvey M, Pantel C, Swain V, Use of an obturator with nCPAP in a premature infant with a cleft lip and palate, pp. 493–497, Copyright (2015), with permission from the Editorial Council for The Journal of Prosthetic Dentistry [2].

There is currently limited literature regarding respiratory support modalities for extremely pre-term neonates with cleft lip and palate. However, the development of the initial obturator used for our patient was inspired by that of Pesun et al. (Figure 3) [2]. In their article, the patient was intubated from birth at 26 weeks until 34 weeks corrected gestational age (weight 2320 g), when non-invasive modalities were expected to be effective. Their patient was extubated with the oral obturator in place to CPAP with a nasal mask interface for 96 hours. Then, ultimately, the patient transitioned off respiratory support.

McMahon et al. described the use of a Laerdal silicone face mask (size 00) incorporated with an Infant Flow System for CPAP delivery in an infant with a bilateral cleft lip and palate, with success on a former 25+1-week gestation infant at 23 days old [10]. A similar device was attempted early on in our patient’s course. However, due to patient activity, the device was unable to be secured properly, resulting in an improper seal and inadequate pressure support.

Airway management in neonates with cleft lip and palate can be challenging, particularly due to difficulty achieving an effective mask seal and facilitating endotracheal intubation. The use of palatal obturators to assist airway management has been described in the literature. Moustafa et al. reported in a randomized controlled trial of infants undergoing first-stage unilateral cleft palate repair that obturator use improved face-mask ventilation and shortened laryngoscopy and induction times, although it did not significantly improve laryngoscopic view [11].

Alternative approaches to respiratory support have also been described in preterm neonates with cleft palates. Eifinger et al. reported stabilization of infants born between 28 and 33 weeks’ gestation using a nasal tube placed in the non-cleft nostril connected to a MediJet generator, allowing time for fabrication of passive orthodontic palatal plates and subsequent transition to nasal CPAP (5-7 cm H₂O) without requiring primary intubation [12].

A combination approach of Eifinger et al.'s first-intention nasal tube connected to a ventilator CPAP/NIPPV, quickly followed by obturator creation by prosthetics teams, would be ideal to limit airway manipulations, prolonged intubation, and subsequent trauma. This sequence of events can then allow for transition to nasal CPAP with an obturator for long-term non-invasive support for the infant to grow and develop.

Various degrees of complications were intervened in during the course of this infant's life. Device dislodgement and oral ulcers were complications that occurred initially but were managed with low-risk interventions, improved greatly with further iterations of the device, and allowed for no further intubation for this patient. Further difficulties arose with frequent bradycardic and desaturation events with device dislodgement, especially during the early iterations of the device. These events were challenging to distinguish at times from other causes (prematurity, sepsis, etc.), and frequent discussions regarding additional caffeine or sepsis workups were held. Ultimately, events improved with adjustments and refitting of the obturator device. Other complications considered were concerns for potential biofilm development on the obturator device, which was mitigated with frequent cleaning.

Overall benefits of this case highlight the use of a custom oral obturator allowing successful extubation for our patient at 29+4 weeks corrected gestation to NIPPV with RAM cannula modality, ultimately preventing a prolonged intubation course (one week total) and the significant complications that can arise. At discharge, the patient was clinically stable on room air, did not meet criteria for a bronchopulmonary dysplasia diagnosis, and had no evidence of intraventricular hemorrhage on serial head ultrasounds [5]. Currently, the patient is developing appropriately for corrected age.

## Conclusions

An early multidisciplinary approach to preterm infants born with cleft lip and palate can be beneficial in providing optimal respiratory care. Specifically, early engagement with the maxillofacial prosthetics team, while often overlooked in the NICU setting, was vital to this patient’s outcome in fashioning different iterations of a custom oral obturator. It is difficult to attribute all of this patient's success to the obutrator device alone, as things like individualized NICU care, lung maturation, and clinical stability also play important roles. However, we hope this case report will provide other care teams with firsthand clinical knowledge and guidance in managing these types of patients. Furthermore, we hope this provides care teams with the willingness and courage to think outside the box in the respiratory care of preterm infants with cleft lip and palate. 
